# Proceedings: Cultured lymphoblastoid lines as a model for leukaemia/lymphoma.

**DOI:** 10.1038/bjc.1975.216

**Published:** 1975-08

**Authors:** C. M. Steel


### CULTURED LYMPHOBLASTOID LINES AS A MODEL FOR LEUKAEMIA/LYMPHOMA

C. M. STEEL, M.R.C. Clinical and Population Cytogenetics Unit, Edinburgh.

With certain notable exceptions, the lymphoblastoid cell lines established from biopsies of Burkitt's lymphoma appear, on cytogenetic and biochemical analysis to be representative of the tumour cells in this condition (Gripenberg, Levan and Clifford, 1969; Klein *et al.*, 1968; Fialkow *et al.*, 1971). Similar lines derived from other sources have the property of indefinite proliferation *in vitro* and may therefore provide a model for the study of at least some aspects of malignancy.

Though little is yet known of the mechanisms involved in the transition from normal lymphocyte to lymphoblastoid cell, there are consistent differences between the two cell types which may provide clues to the nature of the transformation process.

Many established lines have a high level of steroid-binding protein which is also a characteristic of the malignant cells in acute lymphoblastic leukaemia. Preliminary data suggest however that the cytolytic effect of steroids on some lymphoblasts is not dependent solely on the presence of steroid receptors (Gailani *et al.*, 1973; Lippmann, Berry and Thompson, 1974).

Changes in the expression of surface antigens may have an important role in protection of the host against the proliferation of abnormal lymphoid cells *in vivo* (Golub *et al.*, 1972; Steel *et al.*, 1974). The antigenic changes have yet to be fully characterized but HL—A typing reveals both qualitative and quantitative alterations in the expression of transplantation antigens when a cell line becomes established in culture. Similar changes have been detected on leukaemic cells *in vivo*.

Serial studies on lymphoblastoid lines show progressive shifts in the expression of transplantation antigens (Dick *et al.*, 1973). Other characteristics, including karyotype, immunoglobulin synthesis and isoenzyme pattern, also change with time in culture (Steel, McBeath and O'Riodan, 1971; Evans, Steel and Arthur, 1974; Povey *et al.*, 1973). On following genetic markers in individual lines it is clear that the bulk population *in vitro* changes by the emergence of successive waves of clones. If this is a valid model for the behaviour of malignant cells *in vivo* it has important implications for therapy.

### REFERENCES

DICK, H. M., STEEL, C. M. CRICHTON, W. B. & HUTTON, M. M. (1973) International symposium on standardization of HL–A reagents. *Symp. Ser. Immunobiol. Standard*, **18**, 116. Basel: Karger.

EVANS, J., STEEL, M. & ARTHUR, E. (1974) *Cell*, **3**, 153.

FIALKOW, P. J., KLEIN, G., GIBLETT, E. R. GOTHOSKAR, B. & CLIFFORD, P. (1971) *Lancet*, i, 883.

GAILANI, S., MINOWADA, J., SILVERNAIL, P., NUSSBAUM, A., KAISER, N., ROSEN & SHINAOKA, K. (1973) *Cancer Res.*, **33**, 2653.

GOLUB, S. H., SVEDMYR, E. A. J., HEWETSON, J. F. & KLEIN, G. (1972) *Int. J. Cancer*, **10**, 157.

GRIPENBERG, U., LEVAN, A. & CLIFFORD, P. (1969) *Int. J. Cancer*, **4**, 334.

KLEIN, E., KLEIN, G., NADKARNI, J. S., NADKARNI, J. J., WIGZELL, H. & CLIFFORD, P. (1968) *Cancer Res.*, **28**, 1300.

LIPPMANN, M. E., BERRY, S. & THOMPSON, E. B. (1974) *Cancer Res.*, **34**, 1572.

POVEY, S., GARDINER, S. E., WATSON, B., MOWBRAY, S., HARRIS, H., ARTHUR, E., STEEL, C. M., BLENKINSOP, C. & EVANS, H. J. (1973) *Ann. hum. Genet.*, **36**, 247.

STEEL, C. M., MCBEATH, S. & O'RIORDAN, M. L. (1971) *J. natn. Cancer Inst.*, **47**, 1203.

STEEL, C. M., HARDY, D. A., LING, N. R. & LAUDER, I. J. (1974) *Immunology*, **26**, 1013.


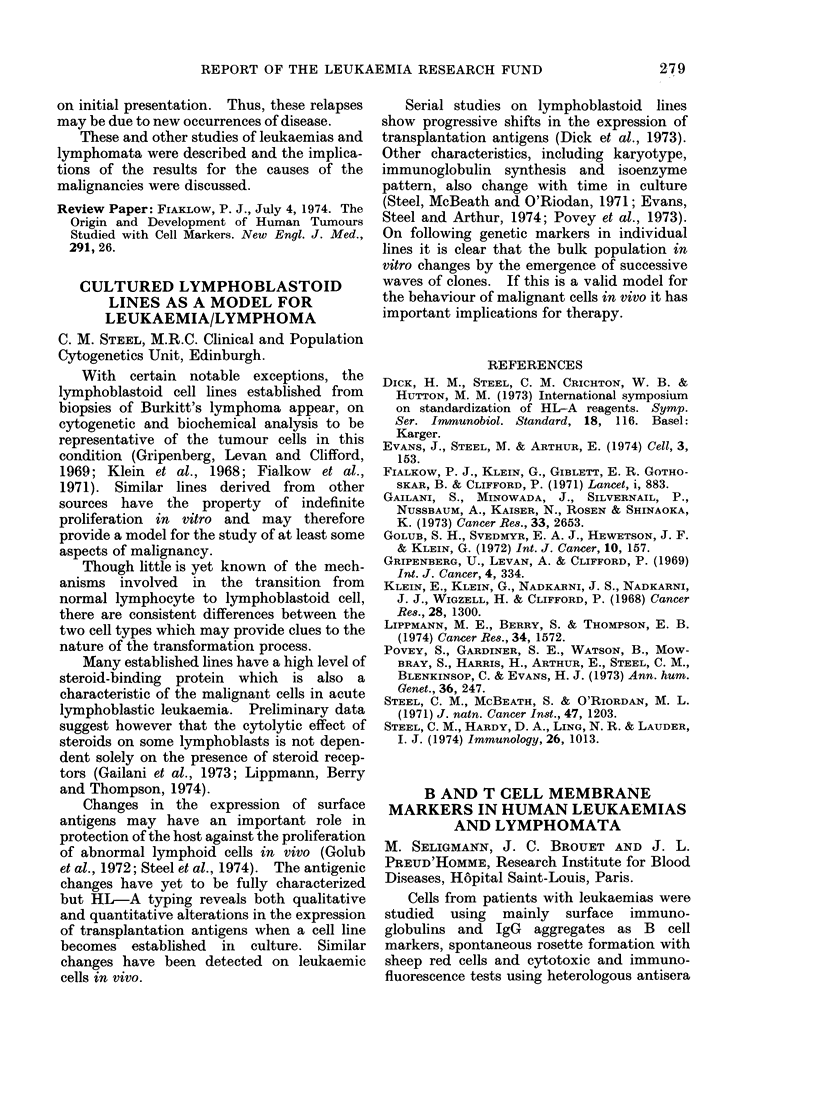


## References

[OCR_00077] Evans J., Steel M., Arthur E. (1974). A hemagglutination inhibition technique for detection of immunoglobulins in supernatants of human lymphoblastoid cell lines.. Cell.

[OCR_00083] Fialkow P. J., Klein G., Giblett E. R., Gothoskar B., Clifford P. (1971). Foreign-cell contamination in Burkitt tumours.. Lancet.

[OCR_00085] Gailani S., Minowada J., Silvernail P., Nussbaum A., Kaiser N., Rosen F., Shimaoka K. (1973). Specific glucocorticoid binding in human hemopoietic cell lines and neoplastic tissue.. Cancer Res.

[OCR_00090] Golub S. H., Svedmyr E. A., Hewetson J. F., Klein G., Singh S. (1972). Cellular reactions against Burkitt lymphoma cells. 3. Effector cell activity of leukocytes stimulated in vitro with autochthonous cultured lymphoma cells.. Int J Cancer.

[OCR_00094] Gripenberg U., Levan A., Clifford P. (1969). Chromosomes in Burkitt lymphomas. I. Serial studies in a case with bilateral tumors showing different chromosomal stemlines.. Int J Cancer.

[OCR_00098] Klein E., Klein G., Nadkarni J. S., Nadkarni J. J., Wigzell H., Clifford P. (1968). Surface IgM-kappa specificity on a Burkitt lymphoma cell in vivo and in derived culture lines.. Cancer Res.

[OCR_00103] Lippman M. E., Perry S., Thompson E. B. (1974). Cytoplasmic glucocorticoid-binding proteins in glucocorticoid-unresponsive human and mouse leukemic cell lines.. Cancer Res.

[OCR_00109] Povey S., Gardiner S. E., Watson B., Mowbray S., Harris H., Arthur E., Steel C. M., Blenkinsop C., Evans H. J. (1973). Genetic studies on human lymphoblastoid lines: isozyme analysis on cell lines from forty-one different individuals and on mutants produced following exposure to a chemical mutagen.. Ann Hum Genet.

[OCR_00117] Steel C. M., Hardy D. A., Ling N. R., Lauder I. J. (1974). The interaction of normal lymphocytes and cells from lymphoid cell lines. VI. Line-directed cytotoxic specificity of lymphocytes activated by autochthonous or allogeneic LCL cells.. Immunology.

[OCR_00113] Steel C. M., McBeath S., O'Riordan M. L. (1971). Human lymphoblastoid cell lines. II. Cytogenetic studies.. J Natl Cancer Inst.

